# THE ASSOCIATION OF SUBCLINICAL HYPERTHYROIDISM WITH FRAILTY

**DOI:** 10.1093/geroni/igz038.2533

**Published:** 2019-11-08

**Authors:** Nalini S Bhalla, Karyne Vinales, Janet Fawcett, Ming Li, Richa Bhattarai, Sherman M Harman

**Affiliations:** 1 Phoenix VA Health Care System, Phoenix, Arizona, United States; 2 Banner University Medical Center-Phoenix, Phoenix, Arizona, United States

## Abstract

The relevance of subclinical hyperthyroidism in the elderly has not been clearly defined. We studied whether the reported association of low TSH with frailty is an indicator of subclinical hyperthyroidism as assessed by free T3 levels. In a retrospective chart review of patients seen between January 2017 and December 2018 at the Phoenix VA Medical Center, we identified 100 patients aged ≥60 years with at least 2 low TSH measurements (<0.5 µIU/ml) and a free T3 measurement within 6 months of the measured TSH and 50 sex- and age-matched controls (TSH 0.5-5.0 µIU/ml). Patients with exogenous or clinical hyperthyroidism were excluded. We used a deficit accumulation approach evaluating 31 factors, to create a frailty index between 0 and 1 for each patient. The higher the FI, the more likely (p<0.001) it was that patients had expired in the interim. Patients with low (0.31±0.11 µIU/mL) vs. normal (1.84±0.84 µIU/mL) TSH had higher mean FI compared to controls (0.25±0.12 vs. 0.15±0.07, p<0.001). TSH significantly predicted frailty score (p<0.0001) independent of age. However, lower TSH was not associated with higher free T3 or free T4 levels. There was a nonsignificant inverse association of free T3 levels with FI (P = 0.09), which disappeared when adjusted for age. Similar to prior studies, low TSH predicted frailty. However, neither free T3 nor free T4 predicted low TSH or frailty index, suggesting that the association of low TSH with frailty is not due to subclinical hyperthyroidism, but perhaps to effects of comorbidities on TSH secretion.

